# Molecular adaptation to calsequestrin 2 (CASQ2) point mutations leading to catecholaminergic polymorphic ventricular tachycardia (CPVT): comparative analysis of R33Q and D307H mutants

**DOI:** 10.1007/s10974-020-09587-2

**Published:** 2020-09-09

**Authors:** Giorgia Valle, Michael Arad, Pompeo Volpe

**Affiliations:** 1 Department of Biomedical Sciences, University of Padova, Viale G. Colombo 3, 35121 Padova, Italy; 2 Leviev Heart Center, Sheba Medical Center, Tel Hashomer and Sackler School of Medicine, Tel Aviv University, Tel Aviv, Israel

**Keywords:** Cathecolaminergic polymorphic ventricular tachycardia, CASQ2 mutations, Degradative pathways, Small heat shock proteins

## Abstract

Homozygous calsequestrin 2 (CASQ2) point mutations leads to catecholaminergic polymorphic ventricular tachycardia: a common pathogenetic feature appears to be the drastic reduction of mutant CASQ2 in spite of normal transcription. Comparative biochemical analysis of R33Q and D307H knock in mutant mice identifies different pathogenetic mechanisms for CASQ2 degradation and different molecular adaptive mechanisms. In particular, each CASQ2 point mutation evokes specific adaptive cellular and molecular processes in each of the four adaptive pathways investigated. Thus, similar clinical phenotypes and identical cellular mechanism for cardiac arrhythmia might imply different molecular adaptive mechanisms.

## Introduction

Homozygous mutations in the cardiac calsequestrin (CASQ2) gene are linked to the recessive form of catecholaminergic polymorphic ventricular tachycardia (CPVT), a life-threatening genetic disease causing dramatic episodes of cardiac arrhythmia and syncope, in the absence of macroscopic structural abnormalities. The genetic background of CPVT is heterogeneous (Faggioni et al. 2012; Al-Hassnan et al. 2013): there are deletions, null mutations as well as missense point mutations which have been studied in some detail, i.e., an arginine residue changed to glutamine at position 33 (R33Q) and an aspartic residue changed to histidine at position 307 (D307H) (Rizzi et al. 2008; Song et al. 2007). Studies of cellular and molecular pathogenesis of CPVT have unravelled adaptive processes that adjust and cope with the presence of a mutant CASQ2 protein and/or with the substantial lack of CASQ2 (Song et al. 2007; Valle et al. 2014). The common pathogenetic feature appears to be the reduction/lack of CASQ2 since the CASQ2 null (KO) model is associated with the CPVT phenotype (Glukhov et al. 2015) .

The present work was undertaken to understand whether two different CASQ2 mutants (R33Q versus D307H) trigger either similar or different adaptive processes. We have identified and compared four different, relevant pathways: (1) activation of ER stress markers such as GRP78, GRP94 and calreticulin (CRT) as well as the compensative and protective protein, Bcl-2, (2) protein degradation pathways, (3) change of Store Operated Ca^2+^ Entry (SOCE) components and (4) activation of small Heat Shock Proteins (sHSPs).

Little is known about the degradative fate of CASQ2; numerous and adaptable degradative pathways might be involved in the heart depending upon age, anatomy, state of health and disease among which are to be recalled the following: the ubiquitin-proteasome system, micro-autophagy, chaperone-mediated autophagy, ER-stress-induced pre-emptive quality control and the calpain system (Kang et al. 2006; Pagan et al. 2013; Nishida et al. 2015; Ghosh and Pattison, 2018).

SOCE is an ubiquitous mechanism linking ER/SR Ca^2+^ depletion to activation of plasma membrane Ca^2+^ channels and includes STIM1, as sensor for Ca^2+^ level in ER/SR and activator of SOCE channels, and SOCE channels, ORAI1 and transient receptor potential channels (TRPCs) (Lopez et al. 2016).

A first defense mechanism, i.e., the HSP system, is deployed by all cells in order to quickly cope with various stress conditions. Based on the molecular mass, different evolutionarily conserved families have been identified; one of them, the sHSPs (Richter et al. 2010), acts as an ATP-independent chaperone able to trap misfolded proteins and avoid aggregation. In heart, Hsp27, Hsp20 and αB-crystallin are the most representative, play various modulatory roles in processes such as protein degradation, cytoskeletal stabilization and apoptosis and are known to be phosphorylated, especially under stress conditions (Bakthisaran et al. 2015).

We have recently studied several adaptive and remodelling mechanisms occuring in hearts of two CPVT mice models, R33Q CASQ2 knock-in (KI) and CASQ2 knock-out (KO) mice, from birth to adulthood (Valle et al. 2014). Here we focus on a third CPVT mouse model, the D307H CASQ2 KI mouse, described by Song et al. (2007), in order to characterize and compare the adaptive processes occurring in the different models.

In the present study, significant differences between the two KI models were identified in each of the four adaptive pathways investigated; thus it is suggested that each CASQ2 point mutation evokes specific adaptive cellular and molecular processes.

## Materials and methods

### Animal models

The animal experiments were approved by Italian Health Ministery (D2784.N.PDJ) and by Tel Aviv University Animal Research Ethics Committee (M-14-063).

Experiments were carried out on male gene targeted C57BL/6j mice, homozygous for R33Q CASQ2 (Valle et al. 2014), D307H CASQ2 (Katz et al. 2013), CASQ2KO (Denegri et al. 2012) compared to age-matched (8 week-old) wild type (WT) (*n* = 4 for each group; body weight 23–30 g).

For D307H KI hearts, some proteins were already studied by others (CASQ2 D307H and CRT in Song et al. 2007; GRP78 and GRP94 in Kalyanasundaram et al. 2010).

R33Q CASQ2 KI mice were genotyped using primers mCASQ2F 5′-CCATGATCTCTATTCTGGAGACTG-3′ and mCASQ2R 5′-CGCAACTTACCTCCAGTACAATCTCC-3′ which produce band size of 290 bp sequenced to determine whether the R to Q mutation exists.

D307H CASQ2 KI mice were genotyped using primers mCASQ2e9F 5′-AAAGTGGCCATTGTTTTCTGTGTCGA-3′ and mCASQ2e9R 5′-GGCAAATTTCTCCTGCCTTCTAAGGA-3′ which produce band size of 774 bp. In order to separate D307H from WT, the PCR product was digested by the restriction enzyme BamHI, which cuts it into two smaller fragments of 271 bp and 500 bp only in the presence of the D307H mutation.

### Whole tissue lysate preparation

Whole hearts were snap-frozen immediately in liquid nitrogen and stored at − 80 °C until protein extraction. Whole heart tissue was homogenized in 3% SDS, 1 mM EGTA with protease inhibitor as previously described (Valle et al. 2014).

### SDS-PAGE and immunoblotting

Quantitative western blotting was carried out on whole heart homogenates from WT, R33Q CASQ2 KI, D307H CASQ2 KI and CASQ2 KO (n = 4 for each group). Equal amounts (100 µg) of protein were subjected to SDS-PAGE and transferred to nitrocellulose membranes by western blot transfer system. Nonspecific binding was blocked with 10% nonfat milk in either PBS or TBST and incubated overnight at 4 °C with antibodies diluited in either PBS or TBST. After washing, membranes were incubated with anti-rabbit (1:10,000) or anti-mouse (1:10,000) secondary antibodies conjugated to either alkaline phosphatase or horseradish peroxidase. In the latter case, visualization was achieved using ECL Western Blotting substrate (Pierce) and images were taken through UVTech instrument (Eppendorf).

Intensity of each band was determined by Scion Image software. Protein-signal densities were normalized to the corresponding actin-signal densities within a linear relationship of antigen concentration versus signal density (Valle et al. 2014).

### Antibodies

Source of specific antibodies: anti-αB-crystallin (mouse, 1:2000), anti-Hsp 20 (rabbit,1:10,000), anti-S16 Hsp-20 (rabbit, 1:500), anti-Hsp27 (rabbit, 1:2000), anti-S82 Hsp27 (rabbit, 1:2000), anti-S59 αB-crystallin (rabbit, 1:2000) and anti-S45 αB-crystallin (rabbit, 1:1000) from Abcam; anti GRP78 (rabbit, 1:500), anti-CASQ2 (rabbit, 1:2000) and anti-CRT (rabbit, 1:1000) from Thermo; anti Bcl-2 (rabbit, 1:1000) and anti-CNX (rabbit, 1:1000) from Santa Cruz; anti-Actin (rabbit, 1:1000), anti-STIM1 (rabbit, 1:2000), anti-TRPC3 (rabbit, 1:500), anti-Orai1 (rabbit, 1:500), anti-EDEM1 (rabbit, 1:1000) and anti-Herp (rabbit, 1:500) from Sigma; anti-Beclin1 (rabbit, 1:5000) and anti-HRD1 (1:1000) from Novus Biologicals; anti-GRP94 (rabbit, 1:1000) from Stressgen; mono- and polyubiquitinated conjugates (mouse, 1:1000) from EnzoLifeScience. Anti Derlin-1 (mouse, 1:1000) was a generous gift of Doriana Sandonà.

### Statistical analysis

Data were expressed as mean ± standard error (SE). Comparisons between means of two groups were performed by unpaired two-tailed Student’s t-test. Comparisons among means of 3 groups were performed by one-way ANOVA Bonferroni’s test. Differences were considered significant at *P < 0.05,**P < 0.01 and ***P < 0.005.

## Results

### Adaptive features of hearts from D307H CASQ2 KI mice

**Fig. 1:**
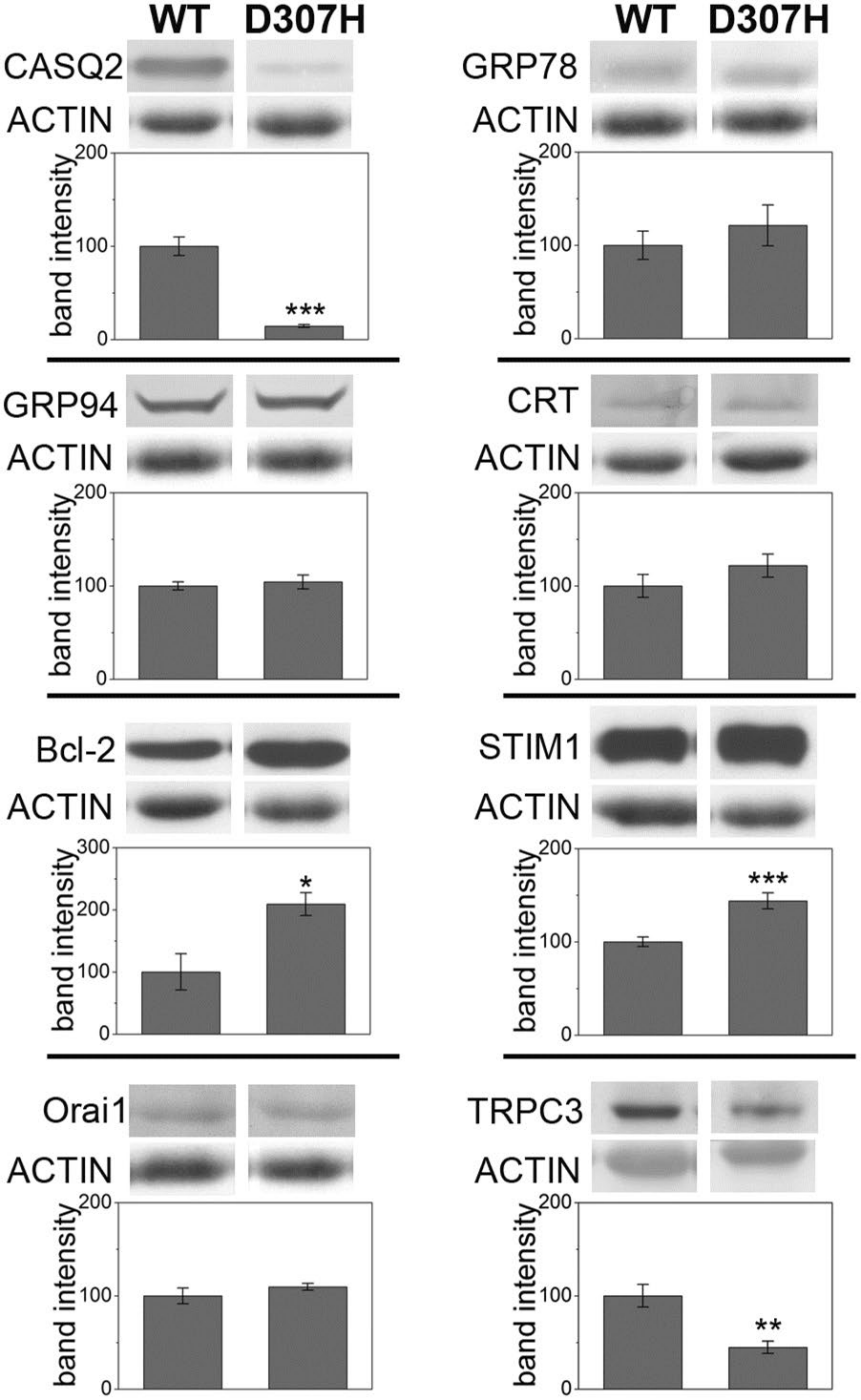
Characterization of D307H knock-in hearts. Relative protein expression of CASQ2, GRP78, GRP94, CRT, Bcl-2, STIM1, Orai1, TRPC3 and actin detected by Western Blot analysis in WT and D307H KI hearts. Average percentages for KI mice are given as mean ± SE (n = 4) and are compared to WT expressed as 100%±SE. *p < 0.05, **p < 0.01, ***p < 0.005, Student’ t-test. Blots are representative images of each group and each experiment was repeated at least thrice

We studied 8 weeks-old D307H KI hearts (Fig. 1) and collected data referable to molecular markers whose expression was found to be altered in R33Q KI and/or KO mice, as compared to WT litter-mates (Valle et al. 2014).

As previously observed in older animals (Song et al. 2007), we confirmed the drastic reduction of expression of D307H CASQ2, as compared to control (14.4 ± 1.5%, *p* < 0.005). R33Q CASQ2 and D307H CASQ2 are missense mutations of CASQ2 and are likely to be post-translationally down-regulated since they might be recognized as misfolded protein inside the ER/SR, thus activating an ER stress response. The conspicuous decrease of D307H CASQ2 protein was not accompanied by up-regulation of ER stress markers, at variance with findings on R33Q CASQ2 (Valle et al. 2014): in fact, the slight increase of GRP78 (121.1 ± 21.9%), GRP94 (104.2 ± 7.5%) and CRT (121.8 ± 12.5%) was not statistically significant. On the other hand, a significant increase was observed in the anti-apoptotic marker Bcl-2 (209.3 ± 18.5%, *p* < 0.05), in accordance with previous observations on both R33Q KI and KO models (Valle et al. 2014).

Alterations in the Store Operated Calcium Entry (SOCE) complex were observed for both R33Q KI and KO models, as judged by expression of STIM1, and Orai1, TRPC3, two SOCE related Ca^2+^ channel (Valle et al. 2014). In D307H KI mice, we observed up-regulation of STIM1 (143.9 ± 8.6%, *p* < 0.005), no difference in Orai1 expression (109.7 ± 3.7) and drastic reduction of the TRPC3 channel (44.8 ± 6.5%, *p* < 0.05), in comparison to WT control.

In conclusion, the D307H KI mouse model shows adaptative mechanisms that markedly differ from the R33Q KI model. In particular, the absence of ER stress activation in D307H KI hearts suggests differences in activation of quality control systems.

### Different key molecules involved in degradative pathways are up-regulated in D307H and R33Q KI models

ER-associated degradation (ERAD) is a quality control system, by which unfolded or misfolded proteins are retrotranslocated from the ER into the cytosol and degraded by the ubiquitin-proteasome system. ERAD occurs in a multistep process including recognition, extraction and ubiquitination of ER proteins. Many ER chaperones are likely to participate in recognition and recruitment of misfolded proteins to the ERAD complex, including GRP78, calnexin (CNX) and EDEM1 (Molinari et al. 2003; Oda et al. 2003). These chaperones deliver substrate to the SEL1L complex, which contains HRD1, homocysteine-induced ER protein (Herp) and Derlin-1 (Hirao et al. 2006; Mueller et al. 2008), for dislocation through the retrograde transport channel. In some cases, ER soluble mutant proteins form aggregates or accumulated in a complex in the ER membrane, so they are not optimal ERAD substrates and are degradated by lysosome via autophagy. Yet, it is not clear whether ER-autophagy or a putative chaperone mediated autophagy (CMA) are involved (Kaushik and Cuervo 2012; Houck et al. 2014; Tao et al. 2019).

To this effect, we studied the expression of CNX, EDEM1, Herp and Derlin-1 as proteins belonging to different steps of the ERAD machinery, in particular Derlin-1 as component of the retrotranslocation machinery. Moreover, Beclin-1, a core component of the class III PI 3-kinase complex, as the initiator of autophagy (Funderburk et al. 2010).

**Fig. 2:**
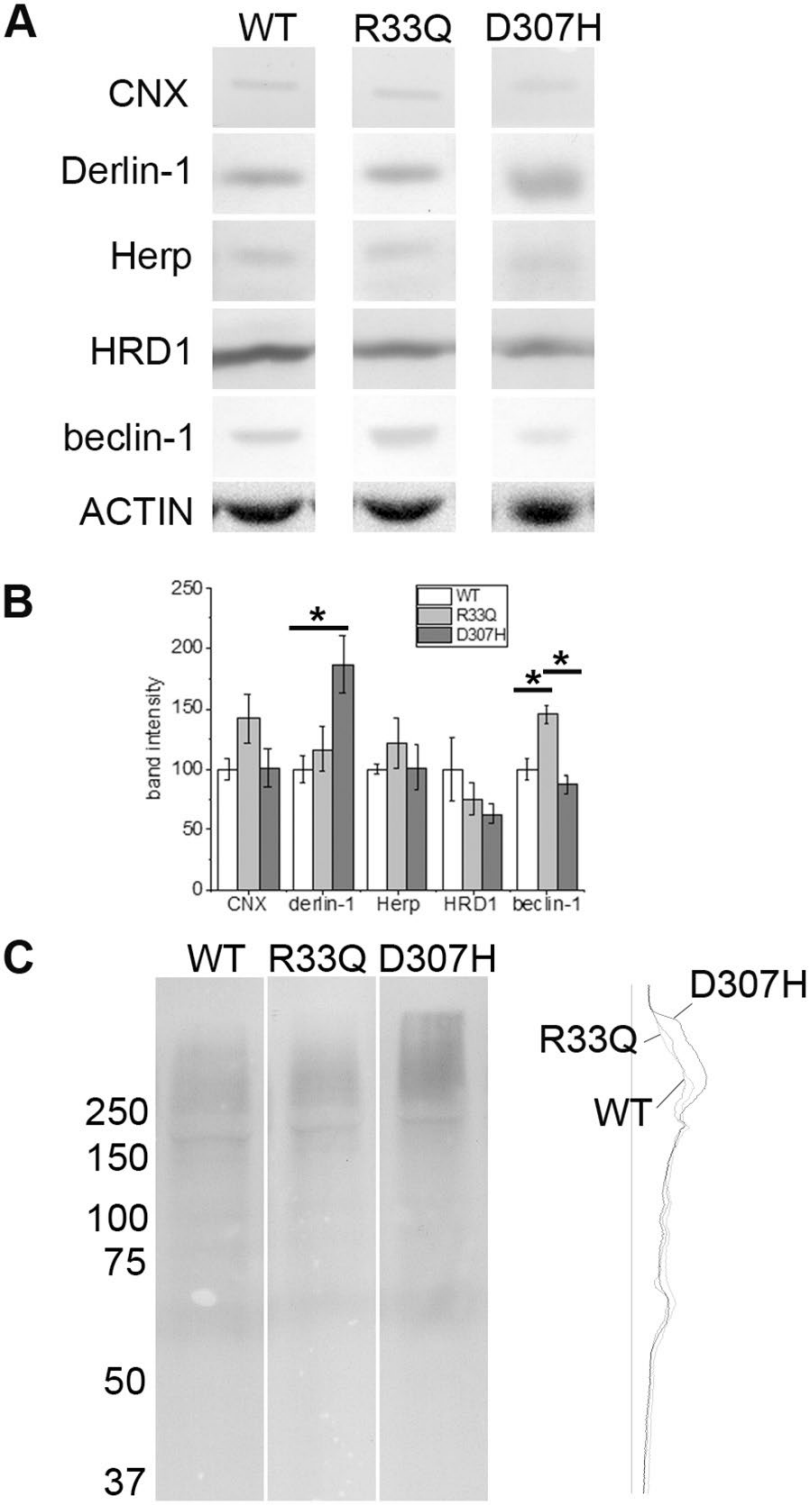
Expression of degradative pathway markers. **a** Relative protein expression of CNX, Derlin-1, Herp, HRD1, Beclin-1 and actin detected by Western Blot analysis in WT, R33Q KI and D307H KI hearts. **b** Average percentages are given as mean ± SE (n = 04) and they are compared to WT expressed as 100% ± SE. Asterisks (*) indicate a p < 0.05 as determined by one-way ANOVA Bonferroni’s test. Blots are representative images of each group and each experiment was repeated at least thrice. **c** Total mono- and poly-ubiquitinated conjugated proteins in WT, R33Q KI and D307H KI hearts detected by Western Blot and on the right, the densitometric profile of the same signal intensities (arbitrary OD) obtained through Scion Image software

As shown in Fig. 2, expression of CNX, Herp and HRD1 was similar in R33Q and D307H KI hearts as compared to WT control; on the contrary, Derlin-1 was significantly up-regulated (187.0 ± 23.9%, *p* < 0.05) in D307H KI hearts but not in R33Q KI hearts (116.3 ± 18.0%). On the other hand, Beclin-1 expression was significantly increased (145.0 ± 7.7%, *p* < 0.05) in R33Q KI hearts but not in D307H KI hearts (87.6 ± 7.7%).

Content of total mono- and poly-ubiquitinated proteins were studied and we observed a mild increase in D307H KI in comparison to WT and R33Q KI (Fig. 2c).

In conclusion, R33Q and D307H KI models show different molecular adaptations, the first involving the autophagy system, the second involving the ERAD pathway, in particular the retrotranslocation machinery towards proteasome.

### Expression of small heat shock proteins in D307H and R33Q KI hearts

sHsps are proteins with molecular mass ranging from 15 to 30 kDa and a conserved α-crystallin domain. sHsps act as molecular chaperones by preventing either aggregation or misfolding of proteins and allow their correct refolding under stress conditions.

**Fig. 3:**
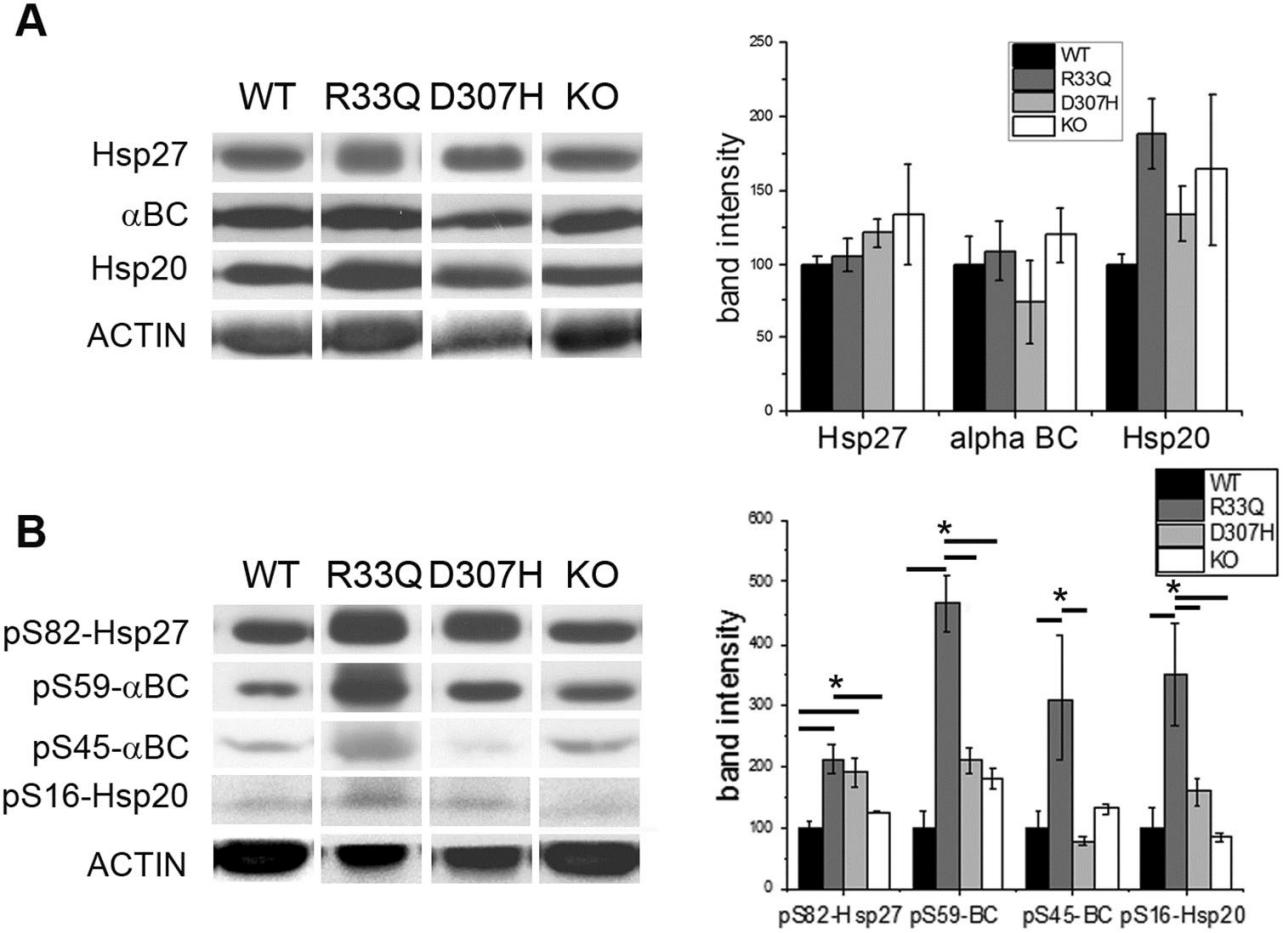
Expresssion of small heat shock proteins. **a** Relative protein expression of Hsp27, αB-crystallin, Hsp20 and actin detected by Western Blot analysis in WT, R33Q KI, D307H KI and KO hearts. On the right, average percentages of R33Q CASQ2, D307H CASQ2 and KO are given as mean ± SE, n = 4, and are compared to WT expressed as 100% ± SE. **b** Relative protein expression of phosphorylated forms of Hsp27 (phosphoSer82), αB-crystallin (phosphoSer59 and phosphoSer45), Hsp20 (phosphoSer16) and actin in WT, R33Q KI, D307H KI and KO hearts. On the right, average percentages of R33Q CASQ2, D307H CASQ2 and KO are given as mean ± SE (n = 4) and are compared to WT expressed as 100% ± SE. Asterisks (*) indicate a p < 0.05 as determined by one-way ANOVA Bonferroni’s test. Blots are representative images of each group and each experiment was repeated at least thrice

We monitored the expression of Hsp27, Hsp20 and αB-crystallin. No differences in sHsps were observed between D307H and R33Q KI hearts (Fig. 3a); on the other hand, their phosphorylation state showed interesting differences, as far as the phosphorylation on S82 of Hsp27, on S59 and S45 of αB-crystallin and on S16 of Hsp20 is concerned. As shown in Fig. 3c increased phosphorylation on S82 of Hsp27 (211.3 ± 23.4%, *p* < 0.05), S59 and S45 of αB-crystallin (465.6 ± 45.4 for S59 and 310.0 ± 101.0% for S45, *p* < 0.05), and S16 of Hsp20 (349.3 ± 84.0% *p* < 0.05) was observed in R33Q KI hearts, whereas increased phosphorylation of S82 Hsp27 (191.1 ± 23.2%, *p* < 0.05) was observed in D307H KI hearts. No alteration whatsoever of phosphorylation state of Hsp27, Hsp20 and αB-crystallin was observed for KO model suggesting that up-regulation of sHsps phosphorylation state was due to the presence of a mutant CASQ2 rather than drastic reduction of CASQ2 content.

In conclusion, R33Q CASQ2 triggers a complex sHSPs-mediated protective response that appears to be different from that evoked by D307H CASQ2.

## Discussion

The goal of this study was to understand how hearts adapt to the presence of mutant CASQ2s comparing two KI mouse models of recessively inherited human CPVT: D307H and R33Q. The present results indicate qualitative differences in induction of ER stress, change of SOCE components, regulation of protein degradation pathways and activation of sHSP protection, and provide new insight in the pathogenesis of CPVT.

Characterization of D307H KI heart with regard to ER stress markers and SOCE components highlights no changes in markers of ER stress, such as GRP78, GRP94 and CRT, up-regulation of Bcl-2 and STIM1 and down-regulation of TRPC3.

In D307H KI mice, a different adaptation of SOCE components was observed; in R33Q KI and KO mice, we recently found up-regulation of STIM1 matched to up-regulation of TRPC channels leading to speculation that SOCE did not represent a complementary process to excitation-contraction coupling but it was crucial in Ca^2+^ handling during diastolic phase (Valle et al. 2014): it was recently shown by Bonilla et al. (2019). For D307H KI mice there was up-regulation of STIM1 with no alteration to ORAI1 and decrease of TRPC3, probably indicating a lower involvement of SOCE.

Previous works (Song et al. 2007; Valle et al. 2014) and the present study report that in adult KI mouse the two mutant proteins, D307H CASQ2 and R33Q CASQ2 (not shown), are well below the WT content level despite normal mRNA expression suggesting that both proteins are subjected to a post-translational control probably caused by up-regulation of protein degradation. Light scattering measurements on the D307H CASQ2 mutant protein suggested that the mutant monomer cannot form a dimer that is properly oriented, subsequently affecting polymerization (Kim et al. 2007). Structural studies have shown that D307H CASQ2 displays similar conformation to WT CASQ2 in absence of calcium whereas it behaves differently at high Ca^2+^ concentrations, indicating a defect in its ability to dynamically handle Ca^2+^ (Kalyanasundaram et al. 2010). Similar results were obtained also for R33Q CASQ2 (Valle et al. 2008), i.e., the mutant monomer can hardly form dimers with the right topology but with different calcium response. Thus, it is entirely plausible that both mutant proteins are not fully working and subsequently recognized and degraded as misfolded proteins. Our results show up-regulation of two different proteins related to degradative pathways: Derlin-1 for D307H KI and Beclin-1 for R33Q KI. Derlin family proteins are components of the ERAD machinery and are required for the retrotranslocation of unfolded/misfolded proteins from the ER to the cytosol for degradation through ubiquitination (Oda et al. 2006). Moreover, it is a key factor of ER-stress-induced pre-emptive quality control, which selectively degrades ER proteins via translocational attenuation through proteasome (Kadowaki et al. 2015). Beclin-1 is a core component of the complex initiator of autophagy; its expression was up-regulated in H9c2 cardiomyoblasts during endoplasmic reticulophagy (Tao et al. 2019). So far no data have been published about CASQ2 degradation in heart neither in health or desease. Katz et al. (2013) using in vivo Bortezomib, a proteasome inhibitor, found a 50% increase of the D307H CASQ2 content in adult KI mice and a partial reversal of the arrhythmic phenotype suggesting that D307H CASQ2 might be preferentially degraded by proteasome in full agreement with our data. On the other hand, as to R33Q KI, autophagy activation is evoked since there are up-regulation of CRT, which was associated to regulation of autophagy signaling acting as LC-3 binding adapter (Yang et al. 2019), and changes in the protein level of Beclin-1 (Yan et al. 2005; Valentim et al. 2006; Hariharan et al. 2011).

In the heart some sHSPs are expressed at high levels, especially Hsp27, αB-crystallin and Hsp20 and have various functions. First, sHSPs act as molecular chaperones to facilitate protein folding, localization, degradation, and function, thereby maintaining proteostasis and preventing various forms of cardiomyocyte damage (Westerheide and Morimoto, 2005). For example, Hsp27 overexpression protects against loss in Ca^2+^ transients and cell shortening in tachypaced HL-1 cardiomyocytes and this protective effect is phosphorylation-dependent (Brundel et al. 2006). The protective effect on Ca^2+^ handling may involve either direct modulation of ion channel function or modulation of specific kinases, resulting in the conservation of ion currents (Christ et al. 2004). Hsp20 promotes the Ca^2+^ cycling in the sarcoplasmic reticulum and enhances the contractile function of the cardiomyocytes (Qian et al. 2011). Some sHSPs were found to interact directly with ion channels, i.e., αB-crystallin with Na^+^ channels (Kashlan et al. 2007). Moreover, sHSPs may prevent cardiomyocyte remodeling via inhibition of proteases, such as calpain. Hsp27 prevents ischemia/reperfusion-induced degradation of the cardiac contractile proteins troponin I and troponin T by interacting with the COOH-terminus and NH_2_-terminus, respectively and preventing calpain cleaving (Lu et al. 2008). Finally, sHSPs are associated with cytoskeletal proteins in a phosphorylation-dependent manner inducing stabilization of cytoskeletal structures and improving resistance to stress conditions such as for αB-crystallin, which binds and stabilizes intermediate filaments and sarcomeric proteins, including actin, desmin and titin (Bullard et al. 2004; Ghosh et al. 2007). Hsp20 promotes the Ca^2+^ cycling in the sarcoplasmic reticulum and enhances the contractile function of the cardiomyocyte (Qian et al. 2011).

For Hsp27 and αB-crystallin, phosphorylation increases chaperone activity and reduces oligomeric size whereas the phosphorylation of Hsp20 at serine 16 was found to protect against cardiac hypertrophy (Qian et al. 2009; Sin et al. 2011).

Very high phosphorylated forms for all the three sHSPs were observed in R33Q KI, whereas only for phosphorylated Hsp27 in D307H KI. In KO hearts we did not find variations in sHSPs content and in phosphorylation forms suggesting that adaptive changes are dependent upon the specific point-mutation and not related to the low CASQ2 level.

The present results suggest that R33Q KI hearts might be totally protected from potential damages and probably against hypertrophy since Hsp20 is more phosphorylated. On the contrary, D307H KI hearts could be more susceptible to hypertrophy and accordingly enlarged atria and ventricles were previously reported at elderly age (Song et al. 2007).

The proposed cellular mechanism for CPVT pathogenesis is based on the aberrant SR Ca^2+^ release activating the electrogenic sarcolemmal NCX to extrude the cytosolic Ca^2+^, causing membrane depolarization and triggering early or delayed after depolarizations (Knollmann et al. 2006; Rizzi et al. 2008). We suggest that the same clinical CPVT phenotype does not imply identical molecular pathogenetic features, depending upon a specific CASQ mutation: key molecules of specific degradative pathways, sHSPs and their phosphorylation status might be interesting topics in understanding cardiac pathophysiology.

## Abbreviations

Bcl-2: B cell lymphoma-2
CASQ2: cardiac calsequestrin
CMA: Chaperone-mediated autophagy
CNX: Calnexin
CRT: Calreticulin
EDEM-1: ER degradation-enhancing alpha-mannosidase-like protein 1
ER/SR: Endoplasmic/sarcoplasmic reticulum
ERAD: Endoplasmic reticulum-associated degradation
GRP78: Glucose-related protein78
GRP94: Glucose-related protein94
sHSP: Small heat shock protein
KI: Knock-in
KO: knock-out
SEL1L: Suppressor/enhancer of Lin12-like
STIM1: Stromal interaction molecule 1
TRPC: Transient receptor potential channel
WT: Wild type

## References

[CR1] Al-Hassnan ZN, Tulbah S, Al-Manea W, Al-Fayyadh M (2013). The phenotype of a CASQ2 mutation in a Saudi family with catecholaminergic polymorphic ventricular tachycardia. Pacing Clin Electrophysiol.

[CR2] Bakthisaran R, Tangirala R, Rao CM (2015). Small heat shock proteins: Role in cellular functions and pathology. Biochim Biophys Acta.

[CR3] Bonilla IM, Belevych A, Baine S, Stepanov A, Mezache L, Bodnar T, Liu B, Volpe P, Priori SG, Weisleder N, Sakuta G, Carnes CA, Radwanski PB, Veeraraghavan R, Gyorke S (2019). Enhancement of cardiac store operated calcium entry (SOCE) within novel intercalated disk microdomains in arrhythmic disease. Sci Rep.

[CR4] Brundel BJ, Henning RH, Ke L, van Gelder IC, Crijns HJ, Kampinga HH (2006). Heat shock protein upregulation protects against pacinginduced myolysis in HL-1 atrial myocytes and in human atrial fibrillation. J Mol Cell Cardiol.

[CR5] Bullard B, Ferguson C, Minajeva A, Leake MC, Gautel M, Labeit D, Ding L, Labeit S, Horwitz J, Leonard KR, Linke WA (2004). Association of the chaperone alphaB-crystallin with titin in heart muscle. J Biol Chem.

[CR6] Christ T, Boknik P, Wohrl S, Wettwer E, Graf EM, Bosch RF, Knaut M, SchmitzW, Ravens U, Dobrev D (2004). L-type Ca2 + current downregulation in chronic human atrial fibrillation is associated with increased activity of protein phosphatases. Circulation.

[CR7] Denegri M, Avelino-Cruz JE, Boncompagni S, DeSimone SA, Auricchio A, Villani L, Volpe P, Protasi F, Napolitano C, Priori SG (2012). Viral gene transfer rescues arrhythmogenic phenotype and ultrastructural abnormalities in adult calsequestrin-null mice with inherited arrhythmias. Circ. Res..

[CR8] Dimauro I, Antonioni A, Mercatelli N, Caporossi D (2018). The role of αB-crystallin in skeletal and cardiac muscle tissues. Cell Stress Chaperones.

[CR9] Faggioni M, Kryshtal DO, Knollmann BC (2012). Calsequestrin mutations and catecholaminergic polymorphic ventricular tachycardia. Pediatr Cardiol.

[CR10] Funderburk SF, Wang QJ, Yue Z (2010). The beclin 1-vps34 complex-at the crossroads of autophagy and beyond. Trends Cell Biol.

[CR11] Ghosh JG, Houck SA, Clark JI (2007). Interactive domains in the molecular chaperone human alphaB crystallin modulate microtubule assembly and disassembly. PLoS ONE.

[CR12] Ghosh R, Pattison JS (2018). Macroautophagy and Chaperone-Mediated Autophagy in heart failure: the known and the unknown. Oxid Med Cell Longev.

[CR13] Glukhov AV, Kalyanasundaram A, Luo Q, Hage LT, Hansen BJ, Belevych AE, Mohler PJ, Knollmann BC, Periasamy M, Gyorke S, Fedorov VV (2015). Calsequestrin 2 deletion causes sinoatrial node dysfunction and atrial arrhythmias associated with altered sarcoplasmic reticulum calcium cycling and degenerative fibrosis within the mouse atrial pacemaker complex1. Eur Heart J.

[CR14] Haghighi K, Bidwell P, Kranias E (2014). Phospholamban interactome in cardiac contractility and survival: a new vision of an old friend. J Mol Cell Cardiol.

[CR15] Hariharan N, Zhai P, Sadoshima J (2011). Oxidative stress stimulates autophagic flux during ischemia/reperfusion. Antioxid Redox Signal.

[CR16] Hirao K, Natsuka Y, Tamura T, Wada I, Morito D, Natsuka S, Romero P, Sleno B, Tremblay LO, Herscovics A, Nagata K, Hosokawa N (2006). EDEM3, a soluble EDEM homolog, enhances glycoprotein endoplasmic reticulum-associated degradation and mannose trimming. J Biol Chem.

[CR17] Houck SA, Ren HY, Madden VJ, Bonner JN, Conlin MP, Janovick JA, Conn PM, Cyr DM (2014). Quality control autophagy degrades soluble ERAD-resistant conformers of the misfolded membrane protein GnRHR. Mol Cell.

[CR18] Kadowaki H, Nagai A, Maruyama T, Takami Y, Satrimafitrah P, Kato H, Honda A, Hatta T, Natsume T, Sato T, Kai H, Ichijo H, Nishitoh H (2015). Pre-emptive quality control protects the ER from protein overload via the proximity of ERAD components and SRP. Cell Reports.

[CR19] Kalyanasundaram A, Bal NC, Franzini-Armstrong C, Knollmann BC, Periasamy M (2010). The calsequestrin mutation CASQ2D307H does not affect protein stability and targeting to the junctional sarcoplasmic reticulum but compromises its dynamic regulation of calcium buffering. J Biol Chem.

[CR20] Kang SW, Rane NS, Kim Sj, Garrison JL, Tauton J, Hedge RS (2006). Substrate-specific translocational attenuation during ER stress defines a pre-emptive quality control pathway. Cell.

[CR21] Kashlan OB, Mueller GM, Qamar MZ, Poland PA, Ahner A, Rubenstein RC, Hughey RP, Brodsky JL, Kleyman TR (2007). Small heat shock protein alphaA-crystallin regulates epithelial sodium channel expression. J Biol Chem.

[CR22] Katz G, Shainberg A, Hochhauser E, Kutzwald-Josefson E, Issac A, El-Ani D, Aravot D, Afek A, Seidman JG, Seidman CE, Eldar M, Arad M (2013). The role of mutant protein level in autosomal recessive catecholamine dependent polymorphic ventricular tachycardia (CPVT2). Biochem Pharmacol.

[CR23] Kaushik S, Cuervo AM (2012). Chaperone-mediated autophagy: a unique way to enter the lysosome world. Trends Cell Biol.

[CR24] Kim E, Youn B, Kemper L, Campbell C, Milting H, Varsanyi M, Kang C (2007). Characterization of human cardiac calsequestrin and its deleterious mutants. J Mol Biol.

[CR25] Knollmann BC, Chopra N, Hlaing T, Akin B, Yang T, Ettensohn K, Knollmann BEC, Horton KD, Weissman NJ, Holinstat I (2006). Casq2 deletion causes sarcoplasmic reticulum volume increase, premature Ca^2+^ release, and catecholaminergic polymorphic ventricular tachycardia. J Clin Invest.

[CR26] Lopez JJ, Albarran L, Gómez LJ, Smani T, Salido GM, Rosado JA (2016). Molecular modulators of store-operated calcium entry. Biochim Biophys Acta.

[CR27] Lu XY, Chen L, Cai XL, Yang HT (2008). Overexpression of heat shock protein 27 protects against ischaemia/reperfusion-induced cardiac dysfunction via stabilization of troponin I and T. Cardiovasc Res.

[CR28] Molinari M, Calanca V, Galli C, Lucca P, Paganetti P (2003). Role of EDEM in the release of misfolded glycoproteins from the calnexin cycle. Science.

[CR29] Mueller B, Klemm EJ, Spooner E, Claessen JH, Ploegh HL (2008). SEL1L nucleates a protein complex required for dislocation of misfolded glycoproteins. Proc Natl Acad Sci USA.

[CR30] Nishida K, Yamaguchi O, Otsu K (2015). Degradation systems in heart failure. J Mol Cell Cardiol.

[CR31] Oda Y, Hosokawa N, Nagata K (2003). EDEM as an acceptor of terminally misfolded glycoproteins released from calnexin. Science.

[CR32] Oda Y, Okada T, Yoshida H, Kaufman RJ, Nagata K, Mori K (2006). Derlin-2 and Derlin-3 are regulated by mammalian unfolded protein response and are required for ER-associated degradation. J Cell Biol.

[CR33] Pagan J, Seto T, Pagano M, Cittadini A (2013). Role of the ubiquitin proteasome system in the heart. Circ Res.

[CR34] Qian J, Ren X, Wang X, Zhang P, Jones WK, Molkentin JD, Fan GC, Kranias EG (2009). Blockade of Hsp20 phosphorylation exacerbates cardiac ischemia/reperfusion injury by suppressed autophagy and increased cell death. Circ Res.

[CR35] Qian J, Vafiadaki E, Florea SM, Singh VP, Song W, Lam CK, Wang Y, Yuan Q, Pritchard TJ, Cai W, Haghighi K, Rodriguez P, Wang HS, Sanoudou D, Fan GC, Kranias EG (2011). Small heat shock protein20 interacts with protein phosphatase-1 and enhances sarcoplasmic reticulum calcium cycling. Circ Res.

[CR36] Richter K, Haslbeck M, Buchner J (2010). The heat shock response: life on the verge of death. Mol Cell.

[CR37] Rizzi N, Liu N, Napolitano C, Nori A, Turcato F, Colombi B (2008). Unexpected structural and functional consequences of the R33Q homozygous mutation in cardiac calsequestrin: a complex arrhythmogenic cascade in a knock in mouse model. Circ Res.

[CR38] Sin YY, Edwards HV, Li X, Day JP, Christian F, Dunlop AJ, Adams DR, Zaccolo M, Houslay MD, Baillie GS (2011). Disruption of the cyclic AMP phosphodiesterase-4 (PDE4)-HSP20 complex attenuates the β-agonist induced hypertrophic response in cardiac myocytes. J Mol Cell Cardiol.

[CR39] Song L, Alcalai R, Arad M, Wolf CM, Toka O, Conner DA, Berul CI, Eldar M, Seidman CE, Seidman JG (2007). Calsequestrin 2 (CASQ2) mutations increase expression of calreticulin and ryanodine receptors, causing catecholaminergic polymorphic ventricular tachycardia. J Clin Invest.

[CR40] Tao T, Wang J, Wang X, Wang Y, Mao H, Liu X (2019). The PERK/Nrf2 pathway mediates endoplasmic reticulum stress-induced injury by upregulation endoplasmic reticulophagy in H9c2 cardiomyoblasts. Life Sci.

[CR41] Valentim L, Laurence KM, Townsend PA, Carroll CJ, Soond, Scarabelli TM, Knight RA, Latchman DS, Stephanou A (2006). Urocortin inhibits Beclin1-mediated autophagic cell death in cardiac myocytes exposed to ischaemia/reperfusion injury. J MOl Cell Cardiol.

[CR42] Valle G, Galla D, Nori A, Priori SG, Gyorke S, de Filippis V, Volpe P (2008). Catecholaminergic polymorphic ventricular tachycardia-related mutations R33Q and L167H alter calcium sensitivity of human cardiac calsequestrin. Biochem J.

[CR43] Valle G, Boncompagni S, Sacchetto R, Protasi F, Volpe P (2014). Post-natal heart adaptation in a knock-in mouse model of calsequestrin2-linked recessive catecholaminergic polymorphic ventricular tachycardia. ExpCell Res.

[CR44] Westerheide SD, Morimoto RI (2005). Heat shock response modulators as therapeutic tools for diseases of protein conformation. J Biol Chem.

[CR45] Wu J, Jiang S, Ding Z, Liu L (2013). Role of the heat shock protein 27 in cardiovascular disease. J Biochem Pharmacol Res.

[CR46] Yan L, Vatner DE, Kim SJ, Ge H, Masurekar M, Massover WH, Yang G, Matsui Y, Sadoshima J, Vatner SF (2005). Autophagy in chronically ischemic myocardium. Proc Natl Acad Sci USA.

[CR47] Yang Y, Ma F, Liu Z, Su Q, Liu Y, Liu Z, Li Y (2019). The ER-localized Ca_2_-binding protein calreticulin couples ER stress to autophagy by associating with microtubuleassociated protein 1A/1B light chain 3. J Biol Chem.

